# Editorial: Chromatographic analytical methods for quantifying newly marketed targeted antitumor drugs

**DOI:** 10.3389/fphar.2023.1308336

**Published:** 2023-10-31

**Authors:** Shashank Kumar

**Affiliations:** Molecular Signaling and Drug Discovery Laboratory, Department of Biochemistry, School of Basic Science, Central University of Punjab, Bathinda, India

**Keywords:** anti-cancer drugs, chromatography, pharmacokinetics, cancer, drug metabolism

Drug metabolism and pharmacokinetics studies are crucial at every stage of the development of new anti-tumour pharmaceuticals. In the body, drugs undergo metabolic transformation, which is a major factor in deciding how safe and effective a medicine is. Several stable metabolites are produced during drug metabolism, the majority of which are pharmacologically inert. However, metabolism can occasionally result in reactive metabolites, which might have negative effects. To produce novel chemical entities with suitable safety profiles, several kinds of drug metabolic studies have been integrated into the drug discovery and development process. This is especially crucial for cancer chemotherapy medications with a limited window of therapeutic action. Conventional anticancer medicines’ high reactivity causes cell toxicity, predicted effects, and main side effects. Special safety and quality precautions are required to deal with these adverse effects. In this regard, analytical approaches for therapeutic drug monitoring of anticancer medicines are crucial (Muhamad and Na-Bangchang, 2020). Pharmacokinetic studies of anticancer medicines in cancer patients are necessary for therapeutic dose and dosing selection (Sabourian et al., 2020). After ingestion, anticancer drugs undergo metabolic pathways that inactivate drug material to produce byproducts or produce the active metabolite for medicinal effects. These routes must be identified by tracking the drug in biological samples using various analytical approaches (Muhamad and Na-Bangchang, 2020; Sabourian et al., 2020). The identification of the anti-tumour drug and/or its metabolites in the pre-clinical sample produces useful information on the optimisation of lead compounds for the best possible pharmacokinetic and pharmacodynamic characteristics. Additionally, in order to reduce potential safety risks brought on by the development of reactive or hazardous metabolites, it will be helpful to identify novel chemical entities based on the metabolites formed. Furthermore, cancer patients receiving multi-drug therapy regimens may benefit from multi-drug quantification techniques in a number of ways, including lowered sample and processing expenses (Sabourian et al., 2020). New drug development, therapeutic drug monitoring, and healthcare professional monitoring are the goals of biological sample anticancer drug monitoring. Human biological samples such as blood, urine, tissue, saliva, and cerebrospinal fluids are essential for monitoring anticancer medications and achieving the goals. Drug quality control relies on drug analysis, a key branch of analytical chemistry. Chromatographic methods are among the most important and reliable analytical methods for measuring anticancer medicines in biologic materials (Muhamad and Na-Bangchang, 2020; Sabourian et al., 2020). The present Research Topic, “*Chromatographic analytical methods for quantifying newly marketed targeted antitumor drugs*,” has gathered four articles, which include original research articles contributed by about 21 potential researchers working in the fields of analytical chemistry and anti-cancer drug discovery.

Bruton’s tyrosine kinase (BTK) protein is expressed at the B cell membrane. The kinase is an important signalling molecule that regulates B cell proliferation, differentiation, and apoptosis. Researchers have developed first-generation BTK inhibitors to treat B-cell-related tumors. Characteristically, the inhibitors were effective and safe compared to traditional chemotherapy for B-cell-related tumours (Wang et al., 2019). It has been reported that the first-generation inhibitor ibrutinib showed intolerance in clinical patients, which resulted in adverse therapy effects (Paydas, 2019). Researchers tried to solve the problem and thus discovered Orelabrutinib as a next-generation BTX inhibitor for the treatment of haematological malignancies (Shirley, 2022). The next step was to generate pharmacokinetic information to determine the safe and tolerable clinical dosage of Orelabrutinib. The original article by Liu et al. reported the development of a chromatographic method for the detection of orelabrutinib plasma concentration. The developed UPLC-MS/MS method showed high recovery, sensitivity, and specificity. The study also recommended the use of orelabrutinib-based pharmacokinetic studies in experimental rats.

Colorectal cancer is one of the major causes of cancer-related mortality worldwide (Sung et al., 2021). Although adjuvant chemotherapy followed by surgery is being practiced to treat advanced or metastatic cancer, the emergence of resistance against clinical anti-colorectal drugs and severe adverse effects necessitated the discovery of newer drugs to at least synergize the available therapeutic agents (Oun et al., 2018; Rejhova et al., 2018). Combining anti-cancer drugs with other therapeutic agents may decrease the drug load and thus minimise the adverse effects, ultimately increasing the therapy outcome. Hao et al. used the supercritical fluid extraction method to extract the active components present in the Angelica sinensis plant and studied the effect of oxaliplatin and A. sinensis extract on colorectal cancer *in vitro* and *in vivo* models. The study showed that the approved anti-cancer drug (oxaliplatin) in combination with the A. sinensis extract produced a potential anti-colon cancer effect compared to a single treatment at a higher dosage. The results suggested that the better potency of oxaliplatin at a lower concentration in combination with the natural product may produce fewer adverse effects. Although the study did not produce any pharmacokinetic data, in the future, the information on oxaliplatin plasma level in an *in vivo* colorectal cancer experimental model will be highly helpful in safer and less toxic dosage calculation in clinical patients.

Liver cancer is a deadly disease with high morbidity and mortality. High proliferation rates of tumour cells necessitate a larger amount of nutrients and energy to meet the physiological needs of the cancer cells. Thus, cancer cells reprogram the metabolic circuit to meet the requirement. The accelerated glycolytic process is one of the reprogramming steps that helps fulfil the energy requirements of cancer cells. The original contribution by Feng et al. reported the effect of Schisantherin A (a plant product) on glucose metabolism in *in vitro* and *in vivo* hepatic cancer experimental models. The compound showed potential anti-hepatocellular potential in the experimental models by decreasing cellular proliferation and migration. The study proposed Schisantherin A as a new therapeutic compound for the treatment of liver cancer. Although the study did not produce any pharmacokinetic data, in the future, the information on Schisantherin A plasma level in an *in vivo* liver cancer model will help in deciding the safer and less toxic dosage for clinical liver cancer patients.

With an expected more than 1.8 million deaths from lung cancer in 2020, lung cancer continues to be the most common cause of cancer-related deaths worldwide. Abemaciclib is an anti-tumour drug used for lung cancer patients. Compared to other drugs (erlotinib), clinical studies showed poor therapy outcomes in terms of overall survival and progression-free survival. In this context, Sun et al. studied the anti-tumour potential of abemaciclib either alone or in combination with gilteritinib (a medication licenced by the FDA for acute myeloid leukaemia) in lung cancer *in vitro* and *in vivo* models. It has been proposed that gilteritinib enhances the cytotoxic potential of abemaciclib through inducing apoptosis, senescence, and the accumulation of vacuoles in lung cancer cells. Abemaciclib, in combination with gilteritinib, inhibited the AKT/Rb pathways in test cells. Further, the study showed G2 phase cell cycle arrest, DNA replication inhibition, and decreased efficiency of DNA repair in abemaciclib and gilteritinib-treated cells. Moreover, in the mouse xenograft model, the drug combination produced a significant reduction in tumour growth with a well-tolerated dose. Results showed that the drug combination (abemaciclib + gilteritinib) is an effective strategy for treating lung cancer, which should be further evaluated in a clinical trial.
